# Arthroscopic Transphyseal ACL Reconstruction With Lateral Extraarticular Tenodesis With Unusual Arthroscopic Meniscal Findings in a Case of an Adolescent Girl Previously Diagnosed With Amniotic Band Syndrome

**DOI:** 10.7759/cureus.54120

**Published:** 2024-02-13

**Authors:** Dhruva Angachekar, Abhay Narvekar, Shivam Patel, Shaswat Shetty

**Affiliations:** 1 Orthopaedic Surgery, Paramount General Hospital & ICCU, Mumbai, IND; 2 Sports Medicine, P. D. Hinduja Hospital & Medical Research Centre, Mumbai, IND; 3 Orthopaedics, Mayo Institute of Medical Sciences, Lucknow, IND; 4 Orthopaedics, P. D. Hinduja Hospital & Medical Research Centre, Mumbai, IND

**Keywords:** lateral extra-articular tenodesis, amniotic band syndrome, meniscal morphology, anterior cruciate ligament reconstruction (aclr), anterior cruciate ligament injury

## Abstract

Amniotic band syndrome (ABS) constriction rings in the lower limb are common. Despite this, there is insufficient literature on anatomical abnormalities in the knee joints of children with ABS. There is an increasing incidence of paediatric anterior cruciate ligament (ACL) injuries recently. ACL reconstruction in this population has an extra dilemma of sparing the physis to prevent growth disturbances. Treating both these conditions simultaneously is a challenge that is rarely encountered. In our literature review, we found no case such as this. As such a case is being described for the first time, we also found certain meniscal anatomical variations on diagnostic arthroscopy. A 12-year-old adolescent Indian girl presented with an ACL tear in her left leg after a school sports injury. She had a known case of ABS constriction bands in both her lower limbs. Her distal femoral and proximal femoral physis was fused on radiographs, so we went ahead with a transphyseal ACL soft-tissue graft reconstruction. On the diagnostic round, we found an anatomical variation of the menisci, which was previously not described since arthroscopy of the knee in an adolescent kid with ABS has not been published in the literature as of yet. These kinds of clinical presentations can become common in the future as more and more kids with ABS take part in recreational sports. In such a scenario, having knowledge about common anatomical variations in the knee of such syndromic patients is essential. While performing ACL reconstructions in this population, we have to be aware of the risk of growth deformities along with vascular and neurological complications, which are added risks with constriction bands around the lower limb.

## Introduction

The number of children participating in hobbies and sports has grown, and this has coincided with an increase in the frequency of anterior cruciate ligament (ACL) injuries [1]. Children who participate in physical activities are at risk of sustaining ACL injuries. It is one of the four ligaments that stabilize the knee joint. The principal function of this ligament is to prevent the tibia from sliding forward in relation to the femur. In addition, the ACL helps prevent tibial rotation, excessive knee extension and varus and valgus knee motions [2]. In this population, there is an added preoperative dilemma of remaining growth and physis sparing so as not to affect the ultimate growth potential of a child. Taking this into consideration, we have four surgical options left with us: physeal-sparing ACL reconstruction, all-epiphyseal ACL reconstruction, partial transphyseal ACL reconstruction and transphyseal ACL ligament reconstruction [3].

Amniotic band syndrome (ABS) is characterized by a variety of congenital abnormalities caused by fibrous bands that are produced from the amniotic sac and constrict one or more regions of the fetus. The other theory is that it is an endogenous vascular anomaly that ultimately causes vascular compromise. Abnormalities in patients with ABS are commonly observed in the lower limbs [4,5]. Despite this, there is insufficient literature on anatomical abnormalities in the knee joints of children with ABS.

We present the case of a 12-year-old girl who presented with an ACL tear in her left leg after a football injury. She had a known case of ABS constriction bands in both her lower limbs.

## Case presentation

**Figure 1:**
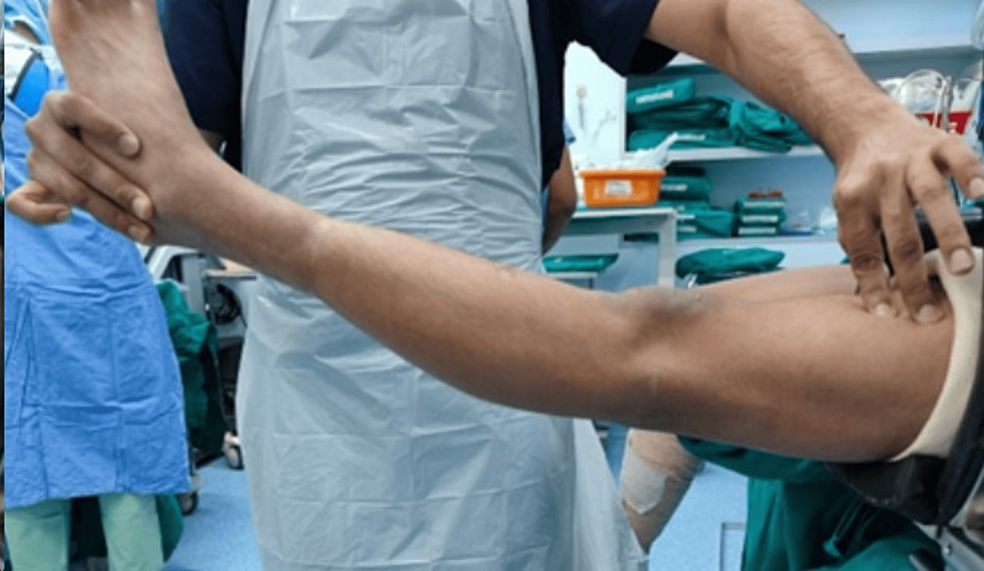
Hyperextension at the knee joint

**Figure 2:**
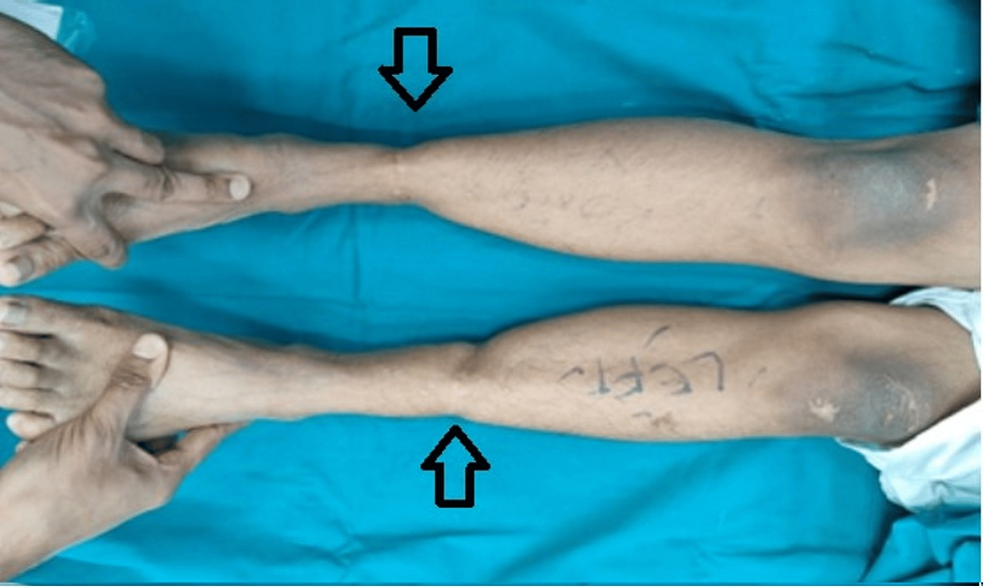
Valgus alignment with bilateral lower limb constriction bands The left knee has an anterior cruciate ligament tear. Arrows point towards the constriction bands on both lower limbs.

A 12-year-old girl presented with pain and instability in her left knee after a football injury at school. On examination, she had a positive Lachman's test and anterior drawer test with no firm end point. The pivot shift test was positive and Grade III. The patient had hyperextension (Figure 1) in her knees and valgus alignment. She had constriction rings over both calves (Figure 2) but was asymptomatic. On other orthopaedic examinations, she had mild thoracolumbar scoliosis.

**Figure 3:**
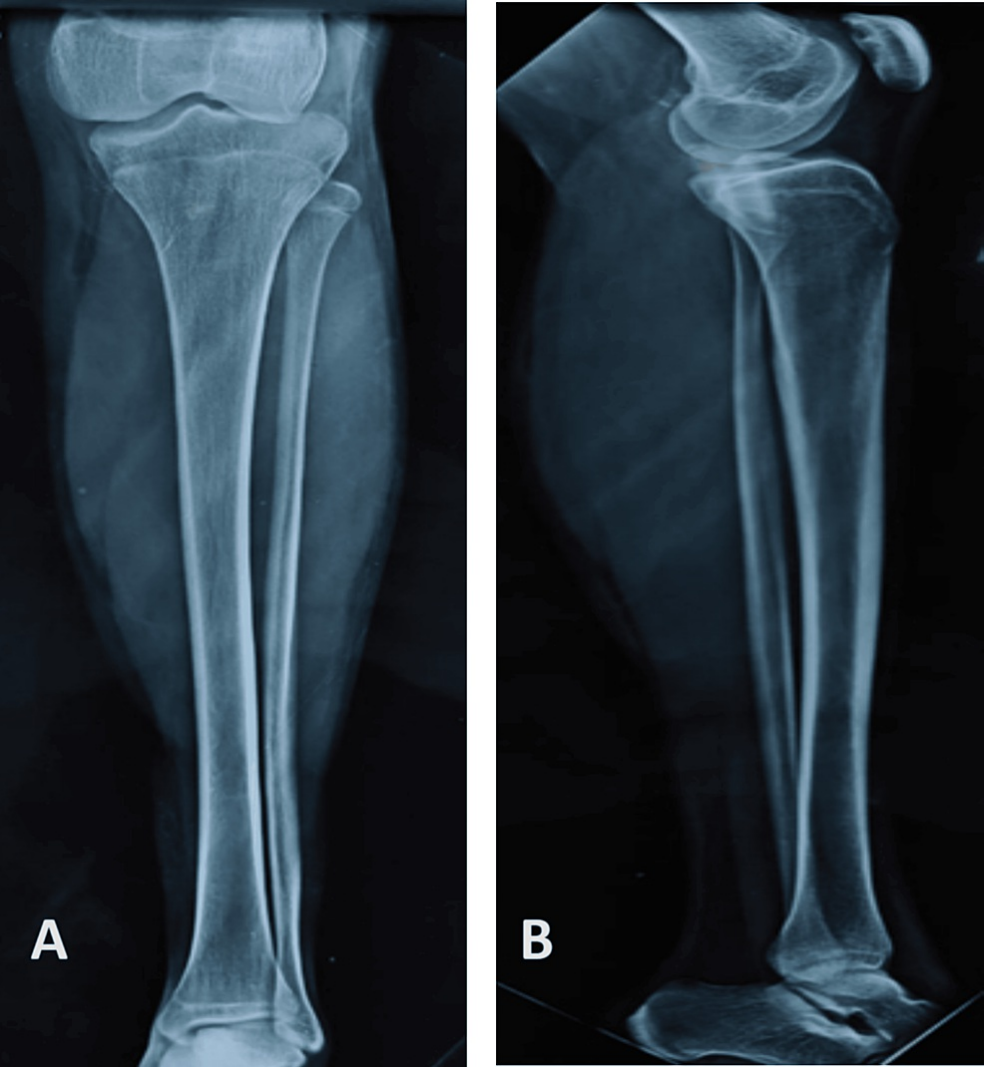
Radiographs of the left lower limb (A) The anteroposterior view shows an almost fused proximal tibial physis. (B) The lateral view shows an almost fused distal femoral and proximal tibial physis with an apparently normal tibial slope.

**Figure 4:**
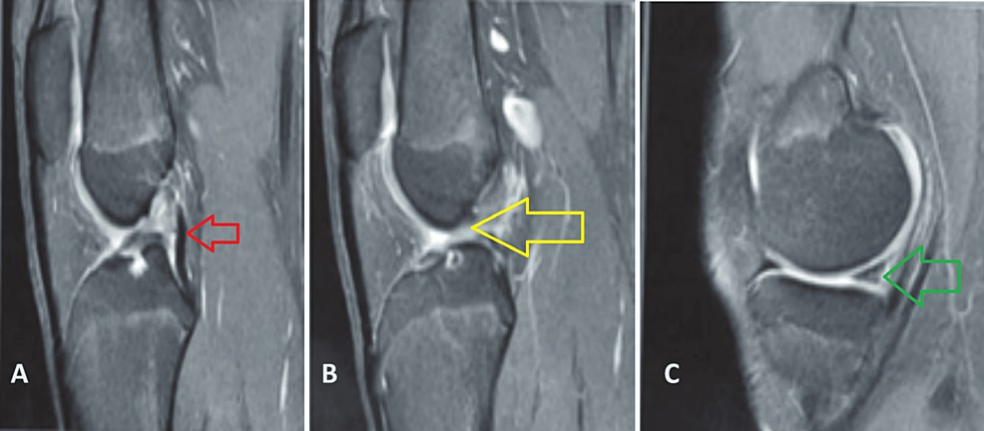
Sagittal MRI sections (A) The red arrow points towards an intact posterior cruciate ligament (PCL). (B) The yellow arrow points towards the torn anterior cruciate ligament (ACL). (C) The green arrow points towards the signal in the posterior horn of the medial meniscus.

**Figure 5:**
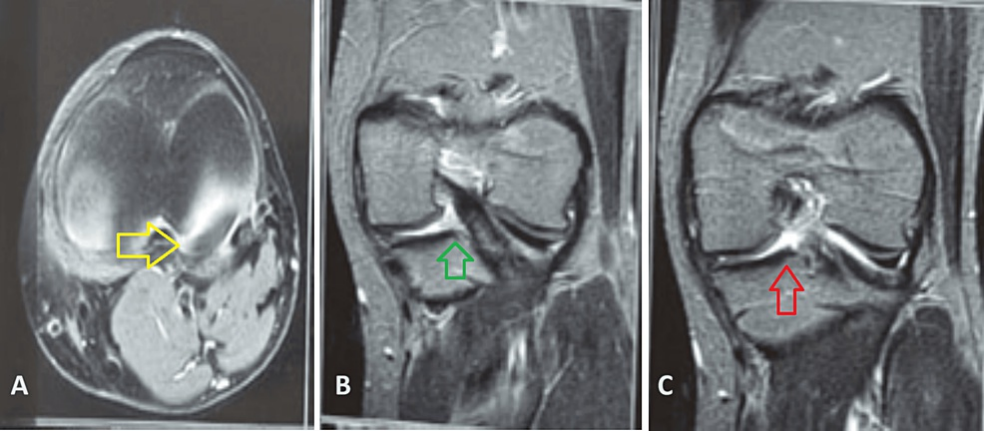
Coronal and axial MRI sections (A) Axial section with the yellow arrow pointing towards the meniscal signal. (B) Coronal section with the green arrow pointing towards no subchondral oedema. (C) Coronal section with the red arrow pointing towards the attached root.

Radiographs showed an almost fused distal femoral and proximal femoral physis (Figure 3). MRI of the left knee showed a complete tear of the ACL in its mid-substance with a signal in the posterior horn of the medial meniscus (Figures 4, 5). Color Doppler studies of the lower limbs were suggestive of a good blood flow distal to the constriction bands. We then decided to go ahead with arthroscopic reconstruction of the ACL. The distal femoral and proximal femoral physis were almost fused in the radiographs, and we determined the bone age based on hand radiographs compared with the Greulich-Pyle atlas. Based on these observations, we decided to proceed with transphyseal ACL reconstruction using a soft-tissue hamstring graft.

**Figure 6:**
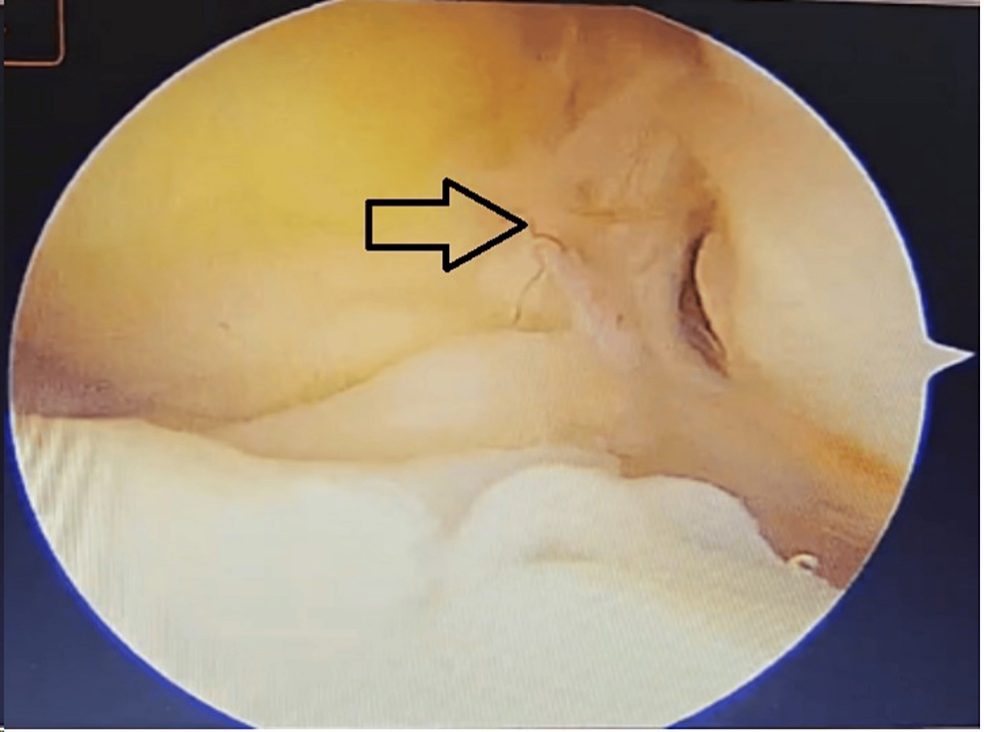
Arthroscopic view of the left knee The arrow points towards a complete tear of the anterior cruciate ligament.

**Figure 7:**
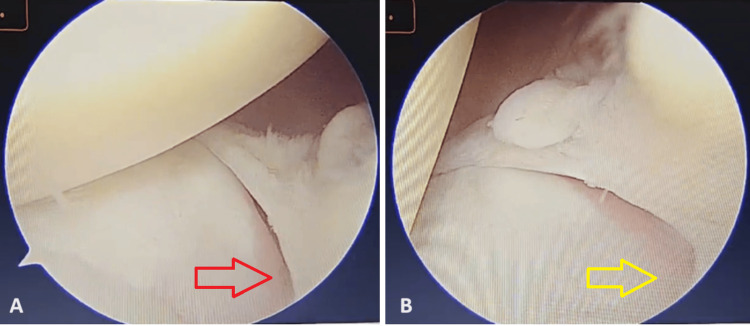
Arthroscopic view of the root of the medial meniscus (A) The red arrow points towards the elevated root of the medial meniscus in the peripheral part. (B) The yellow arrow points towards the elevated root of the medial meniscus in the central part.

**Figure 8:**
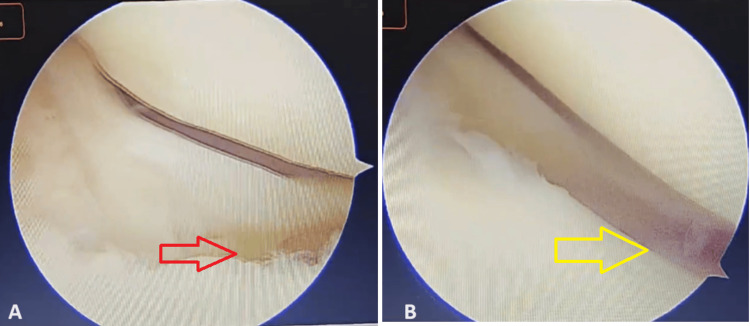
Arthroscopic view of the posterior horns of the medial and lateral meniscus (A) Medial Meniscus: The red arrow points towards the elevated posterior horn. (B) Lateral meniscus: The yellow arrow points towards the elevated posterior horn.

All preoperative workups were performed, and the patient was taken up for surgery. The procedure was performed under general anaesthesia and tourniquet control. The initial diagnostic round showed a complete tear of the ACL ligament (Figure 6). The medial and lateral compartments were then inspected. We took a look at the medial and lateral meniscus. No apparent tear was observed in the menisci. However, we observed an interesting anatomical anomaly. Both meniscal roots were not attached to the tibia (Figure 7), and the posterior horn of both menisci was elevated (Figure 8). Initially, when we observed this on the medial meniscus, we suspected a root tear. However, when we observed similar meniscal anatomy of the lateral meniscus, we realized that this was an anatomical anomaly.

The semitendinosus and gracilis tendons were harvested and prepared by quadrupling each tendon to make an 8 mm eight-strand graft. Subsequently, we removed the ACL stump using a shaver. The femoral tunnel was prepared using a transfemoral approach. The femoral tunnel length was 28 mm, and a tunnel up to 15 mm was drilled with an 8 mm reamer for the graft. On the tibial side, we placed the zig slightly more central on the tibia shin and intra-articularly medial to the anterior horn of the lateral meniscus to get a more central tunnel through the physis. The tibial zig was maintained at 60° to achieve a more vertical tibial tunnel through the proximal tibial physis. This was done with the intention of minimizing the physeal disturbance as much as possible. The tibial tunnel was drilled to a maximum of 8.5 mm. The graft was railroaded through the tunnels and fixed on the femoral side using an endobutton. Tibial fixation was performed using a 15 mm suture washer. There was adequate tension on the graft and no impingement.

We then proceeded to perform lateral extra-articular tenodesis, as she had a Grade III pivot shift. The incision was taken over the lateral aspect of the knee after making proper surface markings for the fibular head, Gerdys tubercle and lateral epicondyle. The tensor fascia lata (TFL) was made visible, and it was freed in a 7 × 80 mm strip above its lower third. Its distal attachment was left intact; however, it was freed at its proximal attachment. After identifying the lateral collateral ligament, the TFL strip was slid beneath it and secured at a location proximal and posterior to the lateral epicondyle. Fixation was performed using a 1.8 mm double-loaded suture anchor. The TFL was closed except at its distal end to prevent lateral compartmental pain. The tourniquet duration was 90 minutes with no vascular or neurological complications in the postoperative period.

**Figure 9:**
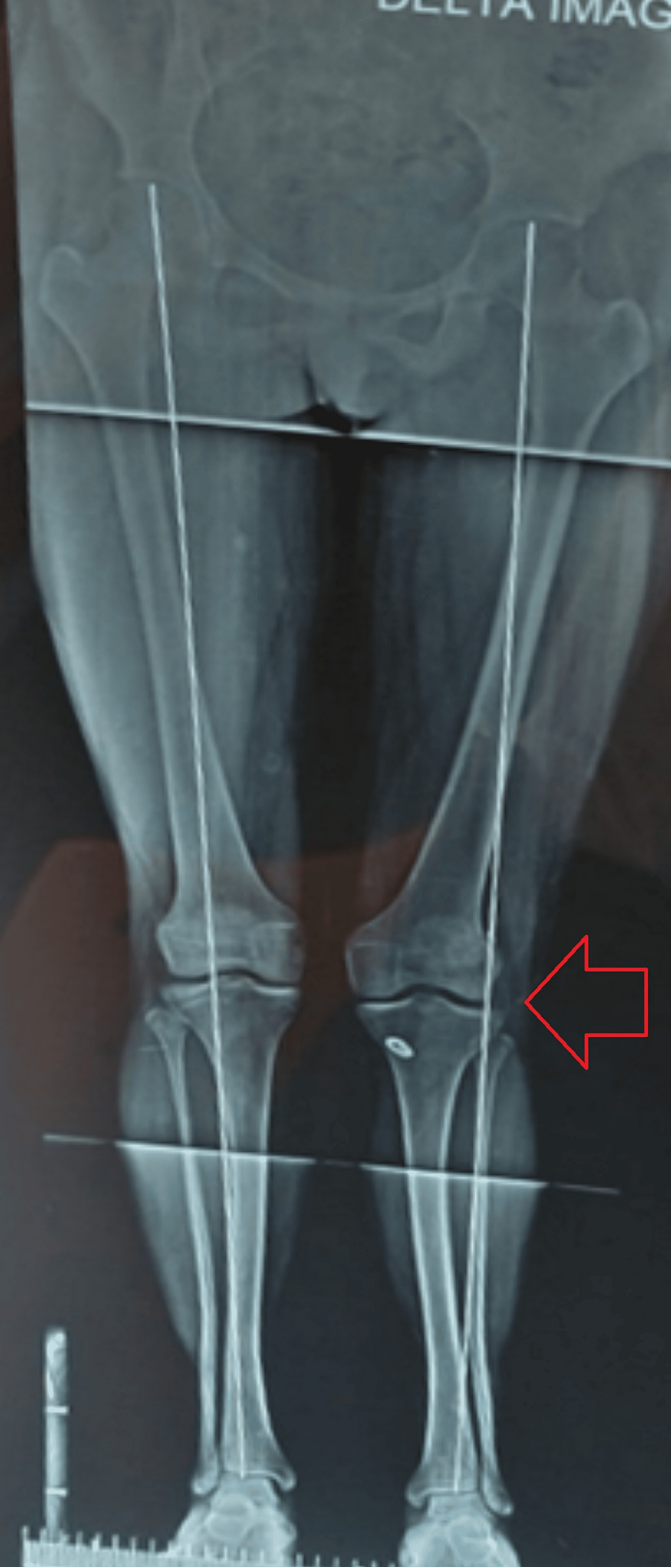
Scanogram of both lower limbs in a standing position with the patella facing forwards The red arrow points towards the weight-bearing axis passing through the lateral aspect of the left knee joint. No postoperative growth deformity was seen.

The patient was discharged from the hospital 24 hours after the surgery and was started on knee bending up to 90° after 48 hours along with static quadriceps, active SLRs and ankle pump exercises. She was called after two weeks for suture removal and was asked to perform a scanogram of both lower limbs with the patella facing forwards in the standing position to have a reference to check for growth disturbances at future follow-ups. The scanogram revealed the weight-bearing axis going through the lateral aspect of the operated knee (Figure 9).

**Figure 10:**
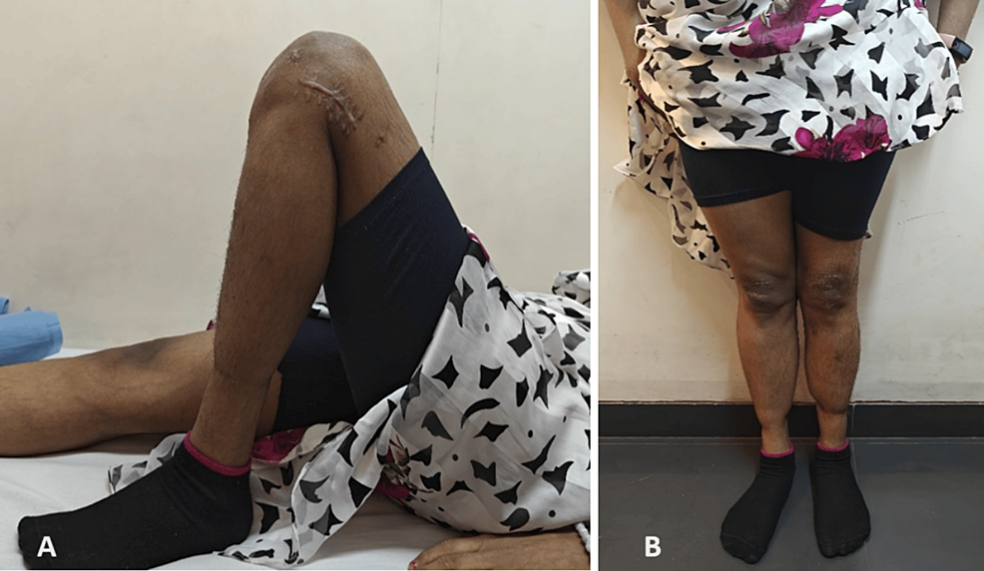
Six months postoperative follow-up clinical pictures (A) Full knee flexion with a healthy lateral knee scar. (B) Clinically acceptable alignment with no growth deformity.

We did regular follow-ups over six months. At the latest follow-up, six months post-surgery, she showed good recovery and progress. There was a firm endpoint on the Lachman and anterior drawer test, while a pivot shift was absent. She had 130° of knee flexion with mild quadriceps wasting on the operated limb. No growth disturbance was noted, and the scar was healthy (Figure 10). She was eventually started on back-to-sports rehabilitation and injury prevention protocol.

## Discussion

The most important finding in this case was a meniscal anatomy variation wherein the meniscal roots appear elevated as would be seen in a case of root tears. Arthroscopic findings of the knee anatomy in cases of ABS are unreported in the literature. ABS is a rare entity in routine pediatric and orthopaedic practice. Research regarding its exact pathogenesis is still ongoing, but there are standardized diagnosis and management protocols for this condition [6]. Congenital malformations of the limbs, such as clubfoot, lymphoedema and syndactyly, are commonly observed in ABS [4,6]. Cases of pseudoarthrosis have also been described in ABS [7]. Most studies on ABS have been conducted in preadolescent children. Our case is unique, as the girl presented with an ACL tear after menarche. During our literature search, we did not find any study related to ACL reconstruction or knee anatomy in patients with ABS. On arthroscopy, we noticed a variation in the meniscal anatomy, which has not been previously described in the literature on ABS.

ACL reconstructions in the adolescent population are increasing with an increasing number of children participating in contact sports. ACL injuries become common with puberty, which is more common in girls than in boys. Intrinsic risk factors for ACL injury include a greater BMI, overpronation of the subtalar joint, generalized ligamentous laxity and a decrease in neuromuscular control of the trunk and lower limbs [8]. Neuromuscular control and ligament laxity with valgus orientation of the limb might have been predisposing factors for ACL injuries in our patient.

Depending on the chronological and skeletal age of patients, surgical arthroscopic ACL reconstruction can be performed using four broad techniques. Physeal-sparing ACL reconstruction avoids creating bone tunnels that cross the physis and is best suited for younger children who still have growth potential left in them. Micheli et al. described a technique of physeal-sparing ACL reconstructions, which provided non-anatomic intra- and extra-articular stability [9].

All-epiphyseal ACL reconstructions offer benefits comparable to the physeal-sparing iliotibial band restoration, but they also restore the anatomic footprint of the ACL. All-epiphyseal ACL reconstruction techniques, including the Anderson, Ganley-Lawrence and Cadasco-Green techniques, have been documented. Each technique has a unique approach to tunnel drilling and fixation [3].

Hybrid transphyseal ACL reconstruction involves tibial transphyseal drilling and fixation in addition to over-the-top femoral physis-sparing graft fixation. Milewski and Nissen reported a more anatomical location of the transphyseal tibial tunnel in conjunction with the entire epiphyseal femoral tunnel [10].

Since most ACL tears occur in adolescents with limited growth, all transphyseal techniques of ACL reconstruction are the most routinely used. Transphyseal ACL repair, when paired with careful patient selection and attention to technical aspects, is a viable therapeutic option for most adolescent patients. Genu valgum and recurvatum can occur due to the use of non-physis-respecting transphyseal techniques. Excessive graft tensioning may lead to recurvatum deformity. We should aim for a vertical trajectory through the distal femoral and the proximal tibial physis as this causes minimum growth disturbance [11]. However, these tunnels are less anatomical and need to be supplemented by an extraarticular stabilising procedure like the lateral extra-articular tenodesis as we have done in our case [12].

Perkins and Willimon in their paper described the technique to choose a graft and reconstruction technique based on the age calculations from a Dmeglio’s chart [3]. For an adolescent female, they chose to do a transphyseal ACL reconstruction using a soft tissue graft and place the femoral and tibial tunnels more centrally. They also advocated long leg radiographs for monitoring growth disturbances for a year, which is similar to the protocol we have followed [3].

Mall et al. in their paper observed that physeal deformities are uncommon when the femoral and tibial physeal violations are small. As a result, in all skeletally immature patients, regardless of physiologic maturity or skeletal age, a modified transphyseal method was used by the authors. With proper techniques, we can keep these to a minimum while performing transphyseal ACL reconstructions in the paediatric population. Thus, they ended up using a semitendinosus with a gracilis hamstring graft, which was passed through more vertically oriented tunnels as the drill holes would be circular rather than oval, which causes the least amount of physeal damage and postoperative growth complications [13].

Francavilla et al. in their study of paediatric meniscal pathologies observed that paediatric meniscal signals appear different from those of adults, which can make the diagnosis on MRI a challenge. Meniscal tears in children, more common in the lateral meniscus, often relate to sports injuries and discoid menisci. Children under 10 showing tears may indicate a discoid meniscus. Meniscal cysts are also commonly seen with peripheral tears in skeletally immature individuals [14]. The signal in the posterior horn of the medial meniscus in our case would have been misleading intraoperatively as the abnormal anatomy of both menisci mimicked a root tear. However, root tears of both menisci simultaneously in the paediatric population are rare, as well as the absence of tibial bone oedema on MRI scans.

Vascular compromise in utero leading to amputations is common in ABS patients. Post-natal vascular compromises are also reported, which cause vascular and lymphatic obstruction. Compressive neurological syndromes are known distal to the constriction band [15]. In our case, despite having a tourniquet time of 90 minutes, we had no neurovascular complications either postoperatively or in the long term.

## Conclusions

ACL tears are rising in the paediatric population with milder lower limb congenital abnormalities who are taking part in sports activities more frequently. They are in fact more predisposed to such injuries because of poor neuromuscular coordination. When undertaking an arthroscopic procedure in such cases, we must be aware that we might encounter abnormal anatomical variations of the ligaments and the menisci because of a syndromic affection of the entire limb. We must also take utmost care in undertaking ACL reconstructions in such cases so as not to increase or develop any deformities already present in the limb. We also need to be aware of vascular and neurological complications that may manifest post-surgery.
